# Effects of bisoprolol in combination with trimetazidine on the treatment of heart failure and concomitant chronic obstructive pulmonary disease

**DOI:** 10.12669/pjms.325.10850

**Published:** 2016

**Authors:** Yuanyuan Ke, Dingli Xu, Minxiong Li, Zenglong Wu, Yongpeng Huang

**Affiliations:** 1 Yuanyuan Ke, Department of Cardiology, Nanfang Hospital, Southern Medical University, Guangzhou 510515, China; 2 Dingli Xu, Department of Cardiology, Nanfang Hospital, Southern Medical University, Guangzhou 510515, China; 3 Minxiong Li, Department of Intensive Care Unit, Gaoming People’s Hospital, Foshan 528500, China; 4 Zenglong Wu, Department of Intensive Care Unit, Gaoming People’s Hospital, Foshan 528500, China; 5 Yongpeng Huang, Department of Intensive Care Unit, Gaoming People’s Hospital, Foshan 528500, China

**Keywords:** Bisoprolol, Trimetazidine, Heart failure, Chronic obstructive pulmonary disease

## Abstract

**Objective:**

To evaluate the effects of bisoprolol combined with trimetazidine on the treatment of heart failure patients having concomitant chronic obstructive pulmonary disease (COPD); in comparison with control group treated with standard therapy only.

**Methods:**

A total of 120 heart failure patients having concomitant COPD were selected and randomly divided into a control group and a treatment group according to different treatment methods (n=60). The control group was given continuous low flow oxygen inhalation and inotropic agents, and their cardiac stress was also reduced. The treatment group was treated with bisoprolol fumarate and trimetazidine in addition to treatment for COPD. For all patients, blood gas analysis and parameters reflecting cardiac function were measured respectively before and after treatment. The respiratory symptoms (cough, sputum, polypnea, gasp, dyspnea), limitation of motion (daily life, household duties, entertainment, sports), disease impacts (social contact, emotion, anxiety) and St. George’s Respiratory Questionnaire (SGRQ) total scores were observed using SGRQ.

**Results:**

The oxygen partial pressure (PaO_2_) and partial pressure of carbon dioxide (PaCO_2_) of the treatment group after treatment were significantly different from those before treatment. After treatment, peak E, E/A and IVEF were increased by 41%, 44% and 16% respectively, but peak A, LVPWT/mm and IVST/mm were significantly reduced. The differences in the respiratory symptoms, limitation of motion, disease impacts and SGRQ total scores were statistically significant compared with those before treatment (P<0.05) and those of the control group (P<0.05).

**Conclusion:**

Combining bisoprolol with trimetazidine in the treatment of heart failure complicating COPD can effectively improve blood gas indices, left ventricular systolic and diastolic functions and the quality of life, thereby alleviating clinical symptoms.

## INTRODUCTION

Heart failure is a common clinical syndrome, and chronic obstructive pulmonary disease (COPD) is a common clinical disease, frequently occurring in association with heart failure. Both entities occurring together has recently become one of the most important cardiovascular diseases.^1^,^2^ Bisoprolol, as a new-generation highly selective β-adrenergic receptor blocker, has been widely used in the treatment of coronary heart disease. However, β-blockers can cause bronchial spasm, resulting in limited application for patients with heart failure complicated with COPD. Trimetazidine is a metabolic drug^3^ that can protect cardiomyocytes and increase their tolerance to effectively improve the cardiac function of patients. Among patients with ischemic cardiomyopathy and heart failure, trimetazidine can be used for myocardial protection, thus effectively reducing the mortality rate.^4^

This study was aimed to explore the clinical efficacy of bisoprolol combined with trimetazidine on COPD patients having heart failure while assessing their quality of life through detecting PaO_2_, PaCO_2_, left ventricular systolic and diastolic functions.

## METHODS

### Selection and grouping of subjects

A total of 120 heart failure patients complicated with COPD who were admitted in our hospital between January 2012 and January 2015 were selected. All the patients had grade II-III cardiac functions based on New York Heart Association (NYHA). There were 68 males and 52 females aged 48-74 years old, (55 ± 12) in average. The heart rate was (55 ± 13) bpm, and the systolic pressure was (134 ± 21) mmHg. All the patients were randomly divided into two groups. The treatment group (n=60) had 32 males and 28 females aged between 45 and 77 years old, with a mean age of 63.2, which included 30 cases of NYHA grade III and grade II respectively. The control group (n=60) had 36 males and 24 females aged between 41 and 83 years old, with a mean age of 65.7, which included 32 cases of NYHA grade III and 28 cases of grade II. There were no significant differences in gender, age or classification of cardiac function between the two groups (P>0.05). The patients with stable hemodynamics, without bronchial asthma, sinus bradycardia, hypotension or severe atrioventricular block were excluded. This study has been approved by the ethics committee of our hospital, and written consent was obtained from all patients.

### Treatment methods

The control group was given continuous low flow oxygen inhalation and inotropic agents, and their cardiac stress was also reduced. Antibiotics, doxofylline for relieving asthma and ambroxol for eliminating phlegm were used when necessary. Based on this, the treatment group was administered with bisoprolol fumarate and trimetazidine. Specific usage: Trimetazidine was orally taken at a dose of 20 mg, 3 times/d. Bisoprolol was taken along with meal in the early morning by an entire tablet with water, and patients should be cautioned against chewing and start taking from a low dose. The regimen for dosage increase is as follows: 1.25 mg in the morning of Week 1; 2.5 mg in the morning of Week 2 if there is a good tolerance; 3.75 mg in the morning of Week 3; 5 mg in the morning of Week 4 to 7; 7.5 mg in the morning of Week 8 to 11; 10 mg in the morning of Week 12, and then the final dose will be a maintenance dose for treatment. The blood pressures of the two groups should be maintained at over 90/60 mmHg, and the heart rates should be kept at above 55 beats/min. ECG showed no atrioventricular block.

### Observation indices before and after treatment

1) Blood gas analysis: The observation indices are PaO_2_ and PaCO_2_; 2) pulmonary function: The percentage of the forced expiratory volume in the first second (FEV_1_) to forced vital capacity (FVC) (FEV1/FVC%); 3) cardiac function: The parameters of left ventricle diastolic and systolic functions (including peak velocity E, A, and E/A ratio, IVEF%) were measured.

### St. George’s Respiratory Questionnaire (SGRQ)

SGRQ, which has been widely used clinically, can evaluate the quality of life in patients with COPD.^5^,^6^ The quality of life of both groups was evaluated using SGRQ before and after treatment in terms of respiratory symptoms (cough, sputum, polypnea, gasp, dyspnea), limitation of motion (daily life, household duties, entertainment, sports), disease impacts (social contact, emotion, anxiety) and SGRQ total scores.

### Statistical analysis

All data were analyzed by SPSS13.0. And expressed as mean ± standard deviation (±s). Inter-group comparisons were performed by one-way analysis of variance. P<0.05 was considered statistically significant.

## RESULTS

### PaO_2_, PaCO_2_, FEV_1_ and FEV_1_% before and after treatment

In the control group, the differences in PaO_2_ and PaCO_2_ were not statistically significant before and after treatment (P>0.05). In the treatment group, after treatment, PaO_2_ was significantly increased by 34% and PaCO_2_ was significantly decreased by 25% compared with those before treatment (P<0.05). After treatment, there were no statistically significant differences in FEV_1_ and FEV_1_% between the treatment group and the control group (P>0.05) (Table-I).

**Table-I:** PaO_2_, PaCO_2_, FEV_1_ and FEV_1_% before and after treatment.

*Group*	*Time*	*PaO_2_ (mmHg)*	*PaCO_2_ (mmHg)*	*FEV_1_ (ml)*	*FEV_1_ %*
Control	Before treatment	55.12±11.23	65.24±4.73	1.24±0.07	67.12
	After treatment	61.04±9.21	64.21±11.21	1.37±0.21	68.21
Treatment	Before treatment	53.07±7.23	67.51±7.73	1.14±0.11	67.41
	After treatment	74.22±3.73*#	53.52±9.43*#	1.31±0.20	68.79

Compared with the same group before treatment,

* P<0.05; compared with the control group at the same time,

# P<0.05.

### Echocardiographic indices before and after treatment

In the control group, there were no statistically significant differences in peak E, peak A, E/A, LVPWT/mm, IVST/mm or IVEF% after treatment compared with those before treatment (P>0.05). In the treatment group, peak E, E/A and IVEF were increased by 41%, 44% and 16% respectively (P<0.05) after treatment compared with those before treatment, whereas peak A, LVPWT/mm and IVST/mm were significantly lowered (P<0.05). After treatment, peak E was significantly increased (by 30%) in the treatment group compared with that of the control group (Table-II).

**Table-II:** Echocardiographic indices before and after treatment (χ̄±s).

*Group*	*Time*	*Peak E (cm/s)*	*Peak A (cm/s)*	*E/A*	*LVPWT (mm)*	*IVST (mm)*	*FEV_1_ %*
Control	Before treatment	42±11	6±12	0.65±0.19	9.71±1.19	12.25±1.37	59±6
	After treatment	52±10	69±11	0.79±0.11	9.49±0.71	0.99±1.32**	163±23
Treatment	Before treatment	45±9	81±17	0.74±0.32	10.65±1.32	12.64±0.98	57±10
	After treatment	75±10*#	54±9*#	1.30±0.41*#	9.59±1.52**	10.31±1.67*	68±8*

Compared with the same group before treatment,

* P<0.05,

** P<0.01; compared with the control group at the same time,

# P<0.05.

### SGRQ scores before and after treatment

There were no statistically significant differences in respiratory symptoms, limitation of motion, disease impacts or SGRQ total scores between the treatment group and the control group before treatment (P>0.05). In the treatment group, the above differences were statistically significant before and after treatment and from those of the control group (P<0.05) (Table-III).

**Table-III:** SGRQ scores before and after treatment.

*Item*	*Control*		*Treatment*	

	*Before treatment*	*After treatment*	*Before treatment*	*After treatment*
Respiratory symptom	66±11	61±12	65±12	51±13*#
Limitation of motion	61±9	57±13	59±11	43±10*#
Disease impact	67±12	65±11	57±9	53±12*#
SGRQ total score	55±13	54±12	54±12	40±11*#

Compared with the same group before treatment,

* P<0.05; compared with the control group at the same time,

# P<0.05.

## DISCUSSION

A large number of evidence-based medical studies have proved that in the development of heart failure, neurohumoral factors, ventricular remodeling, diastolic dysfunction and change of humoral factors play vital roles in the compensatory mechanism of the myocardium. Heart failure complicated with COPD may exacerbate hypoxia, cause ventilation/perfusion defects, and increase the blood oxygen diffusion barrier, which lead to various degrees of hypoxemia and hypercapnia, culminating in respiratory failure. The increased activity of the cardiac decompensation mechanism of respiratory failure patients (renin-angiotensin-aldosterone system) and the sympathetic nervous system cause myocardial hypertrophy and ventricular remodeling, resulting in a vicious cycle.^7^,^8^ Currently, both the Heart Failure Society of America guidelines and Chinese heart failure guidelines^9^ consider selective β-receptor blockers can be used as standard anti-heart failure treatment, but β-receptor blockers with different targets also have some differences in their clinical effects, of which β1 receptors are mainly distributed in the heart, and β2 receptors in the peripheral vascular, liver, skeletal muscle, pancreas, urinary and reproductive system, adipose tissue and bronchi. Activation of β2 receptors can maintain normal trachea relaxation and glucolipid metabolism. When these receptors are blocked, glucolipid metabolism will be affected, causing bronchial spasm.^10^,^11^ As COPD patients with heart failure usually have poor conditions in the respiratory system, it is crucial to choose appropriate β receptor blockers.

Bisoprolol is a highly selective β-adrenoceptor antagonist whose selectivity to β1/β2 receptors is about 120:1, without intrinsic sympathomimetic activity or membrane-stabilizing activity. Compared with its similar drug carvedilol, bisoprolol has better effects on reducing left ventricular hypertrophy and improving diastolic function as well as a greater advantage in the control of heart rate, with fewer adverse reactions and higher safety.^12^,^13^

Beta-blockers are a class of drugs used to control symptoms of heart failure that are made worse by certain hormones.^14^ β-Blockers have been used routinely to treat patients with stage A heart failure (HF) with hypertension. Recent controversy regarding the detrimental effects that some β-blockers have on metabolic parameters has raised inappropriate concerns about the use of any β-blocker for diabetes. β-Blockade is standard therapy for the patient with stage B HF who has had a myocardial infarction, but limited data is available concerning use in asymptomatic patients with left ventricular dysfunction. Additionally, β-blockers are part of the core therapy for stage C HF and selected patients with stage D HF.^15^,^16^

Bisoprolol has a high affinity to β1 receptors of bronchi and vascular smooth muscle, but low affinity to their β2 receptors and those regulating metabolism. Therefore, bisoprolol usually cannot affect airway resistance or the metabolic effects of β-receptor regulation. Moreover, it still has β-receptor selectivity at the time of beyond therapeutic doses. In addition, coronary artery disease patients with COPD well tolerate β-receptor blockers with high selectivity,^17^ and highly selective β-adrenoceptor antagonist barely affects the airway of COPD patients.^18^ Trimetazidine is a new drug regulating myocardial metabolism, which can inhibit the oxidation of myocardium, inhibit free oxygen molecules and promote the aerobic metabolism of glucose, thereby reducing myocardial oxygen consumption to further protect cardiomyocytes. The cardiomyocytes of patients with ischemic cardiomyopathy often undergo acidosis because of ischemia, while trimetazidine can effectively reduce ischemic acidosis symptoms of cells, inhibit the aggregation of sodium and calcium ions, and effectively increase the mitochondrial activity.^19^ Furthermore, trimetazidine can also inhibit oxygen free radicals in blood and exert protective effects on the myocardium to further reduce the damage in cardiomyocytes due to oxygen consumption, so that enhanced glucose oxidation can optimize cellular energy processes to maintain appropriate energy metabolism upon ischemia. Trimetazidine does not affect the body’s hemodynamics while achieving anti-ischemic effects.^20^

We herein studied the effects of bisoprolol combined with trimetazidine on heart failure having concomitant COPD. The blood gas indices of the treatment group were significantly improved, of which PaO_2_ was increased significantly and PaCO_2_ was decreased markedly. Meanwhile, the left ventricular diastolic function was improved obviously, and the left ventricular systolic function was enhanced. Additionally, the respiratory symptoms, limitation of motion, disease impact, SGRQ total scores of the treatment group all exceeded those before treatment. Hence, combining bisoprolol with trimetazidine is a safe and effective regimen with good compliance.

Others clinical trials have consistently shown the benefits of beta-blocker treatment in patients with chronic HF. As a result, bisoprolol, carvedilol, and metoprolol succinate are now indicated for the treatment of all patients with chronic HF who do not have major contraindications. Bisoprolol is demonstrated to improve survival in an outcome trial. In the Cardiac Insufficiency Bisoprolol Study II, all-cause mortality and sudden death were reduced in patients treated with bisoprolol compared with those on placebo (11.8% vs 17.3%; p < 0.0001 and 3.6% vs 6.3%, p < 0.002; respectively) regardless of age, NYHA functional class, and co-morbidities.^21^ A systematic literature search was conducted to identify randomized controlled trials of trimetazidine for HF. Trimetazidine therapy was associated with a significant improvement in left ventricular ejection fraction in patients with both ischemic and non-ischemic HF, trimetazidine might be an effective strategy for treating HF.^22^,^23^
